# GTPase splice variants RAC1 and RAC1B display isoform-specific differences in localization, prenylation, and interaction with the chaperone protein SmgGDS

**DOI:** 10.1016/j.jbc.2023.104698

**Published:** 2023-04-12

**Authors:** Olivia J. Koehn, Ellen Lorimer, Bethany Unger, Ra’Mal Harris, Akansha S. Das, Kiall F. Suazo, Shelby A. Auger, Mark D. Distefano, Jeremy W. Prokop, Carol L. Williams

**Affiliations:** 1 Department of Pharmacology and Toxicology, Medical College of Wisconsin, Milwaukee, Wisconsin, USA; 2 Department of Pediatrics and Human Development, College of Human Medicine, Michigan State University, Grand Rapids, Michigan, USA; 3 Department of Chemistry, University of Minnesota, Minneapolis, Minnesota, USA; 4 Department of Pharmacology and Toxicology, Michigan State University, East Lansing, Michigan, USA

**Keywords:** Ras-related C3 botulinum toxin substrate 1 (RAC1), RAC1B, small GTPase, protein isoprenylation, protein–protein interaction, nuclear transport, click chemistry, SmgGDS, RAP1GDS1

## Abstract

Identifying events that regulate the prenylation and localization of small GTPases will help define new strategies for therapeutic targeting of these proteins in disorders such as cancer, cardiovascular disease, and neurological deficits. Splice variants of the chaperone protein SmgGDS (encoded by *RAP1GDS1*) are known to regulate prenylation and trafficking of small GTPases. The SmgGDS-607 splice variant regulates prenylation by binding preprenylated small GTPases but the effects of SmgGDS binding to the small GTPase RAC1 *versus* the splice variant RAC1B are not well defined. Here we report unexpected differences in the prenylation and localization of RAC1 and RAC1B and their binding to SmgGDS. Compared to RAC1, RAC1B more stably associates with SmgGDS-607, is less prenylated, and accumulates more in the nucleus. We show that the small GTPase DIRAS1 inhibits binding of RAC1 and RAC1B to SmgGDS and reduces their prenylation. These results suggest that prenylation of RAC1 and RAC1B is facilitated by binding to SmgGDS-607 but the greater retention of RAC1B by SmgGDS-607 slows RAC1B prenylation. We show that inhibiting RAC1 prenylation by mutating the CAAX motif promotes RAC1 nuclear accumulation, suggesting that differences in prenylation contribute to the different nuclear localization of RAC1 *versus* RAC1B. Finally, we demonstrate RAC1 and RAC1B that cannot be prenylated bind GTP in cells, indicating that prenylation is not a prerequisite for activation. We report differential expression of RAC1 and RAC1B transcripts in tissues, consistent with these two splice variants having unique functions that might arise in part from their differences in prenylation and localization.

Small GTPases are critical signaling proteins that are regulated by guanine nucleotide binding state, as well as by prenylation and subcellular localization. The small GTPase RAC1 is a member of the Rho family of small GTPases and participates in signaling pathways that regulate cytoskeletal organization, cell proliferation and survival, and gene transcription (1, 2). The human *RAC1* gene encodes two major splice variants that generate proteins referred to as RAC1 and RAC1B (Table 1 and Fig. 1*A*). RAC1B differs from RAC1 only by the presence of a 19-amino acid insertion encoded by exon 3b, immediately following the Switch-II region (3, 4, 5). Compared to RAC1, RAC1B has distinct downstream signaling and altered activity, which are likely to have implications for normal cellular signaling and pathophysiology.

**Table 1:** Description of proteins encoded by transcripts from the *RAC1* and *RAP1GDS1* genes

Common protein name	UniProt identifier	Transcript identifiers	Protein size	Structural elements	Notes
*RAC1* gene products					
RAC1 (9, 15, 16, 58)	P63000-1	RAC1-201 ENST00000348035.9	192 amin o acids	Figure 1*A*	This isoform is the canonical sequence
RAC1B (9, 15, 16)	P63000-2	RAC1-202 ENST00000356142.4	211 amino acids	Figure 1*A*	Identical to P63000-1 but has a 19 amino acid insert
*RAP1GDS1* gene products					
SmgGDS-607 (27, 28, 29, 30, 31, 33, 35)	P52306-1	RAP1GDS1-205 ENST00000408927.8	607 amino acids	Figure 1*D*	This isoform is the canonical sequence: It has 13 ARM domains labeled A - M
SmgGDS-558 (27, 28, 29, 30, 31, 33, 35)	P52306-2	RAP1GDS1-204 ENST00000408900.7	558 amino acids	Figure 1*D*	Identical to P52306-1 but lacks ARM C
SmgGDS-559 (32)	P52306-3	RAP1GDS1-203 ENST00000380158.8	559 amino acids	Figure 1*D*	Identical to P52306-2, but has an additional alanine at residue 2
SmgGDS-608*var* (suggested)	P52306-4	RAP1GDS1-206 ENST00000453712.6	607 amino acids	Figure 1*D*	Identical to P52306-5 but lacks the alanine found at residue 434 in P52306-5
SmgGDS-608 (32)	P52306-5	RAP1GDS1-202 ENST00000339360.9	608 amino acids	Figure 1*D*	Identical to P52306-1 but has an additional alanine at residue 2
SmgGDS-516 (suggested)	P52306-6	RAP1GDS1-201 ENST00000264572.11	516 amino acids	Figure 1*D*	Has unique ARM domain organization
SmgGDS-253 (suggested)	H0Y8M2	RAP1GDS1-214 ENST0000050901.5	253 amino acids	Figure 1*D*	Has unique ARM domain organization
SmgGDS-158A (suggested)	D6REZ0	RAP1GDS1-216 ENST00000511212.5	158 amino acids	Figure 1*D*	Has unique ARM domain organization
SmgGDS-158B (suggested)	D6RB97	RAP1GDS1-219 ENST00000514122.5	158 amino acids	Figure 1*D*	Has unique ARM domain organization
SmgGDS-147 (suggested)	D6RHH8	RAP1GDS1-211 ENST00000508213.5	147 amino acids	Figure 1*D*	Has unique ARM domain organization
SmgGDS-122 (suggested)	D6RHZ7	RAP1GDS1-213 ENST00000509011.5	122 amino acids	Figure 1*D*	Has unique ARM domain organization
SmgGDS-101 (suggested)	U3KQJ4	RAP1GDS1-220 ENST00000514139.2	101 amino acids	Figure 1*D*	Has unique ARM domain organization

**Figure 1:**
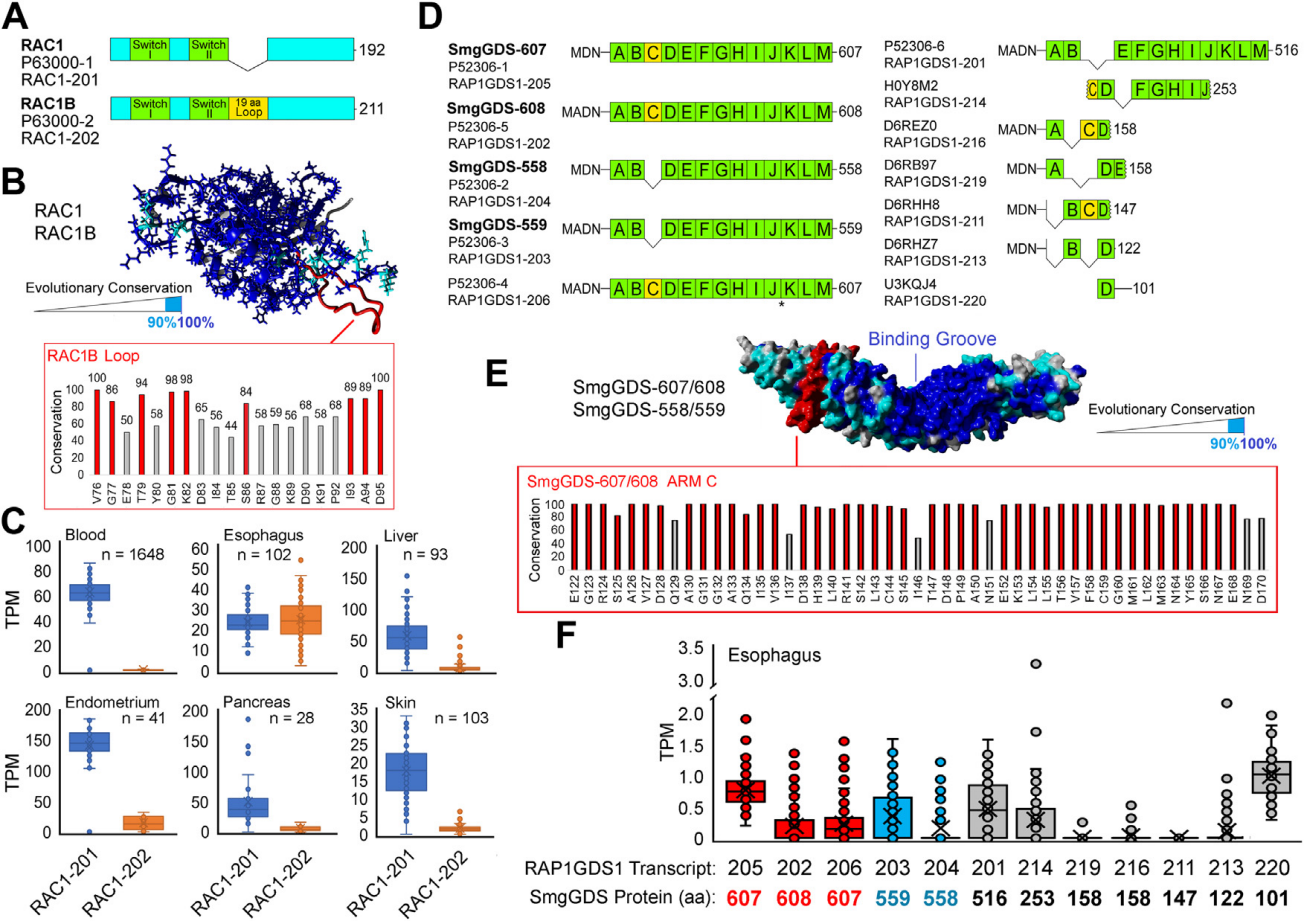
**Evolution and expression of RAC1 and SmgGDS isoforms.***A*, the two proteins encoded by spliced transcripts from the human *RAC1* gene are shown. The *RAC1-201* transcript encodes a 192 amino acid protein, called RAC1 (protein identifier P63000-1), and the *RAC1-202* transcript encodes a 211 amino acid protein, called RAC1B (protein identifier P630000-2). RAC1B differs by the insertion of 19 amino acids that form a loop, following the switch II region (*yellow highlight*). *B*, evolution of 204 RAC1 sequences identified on the models of RAC1. Amino acids conserved 100% are shown in *blue*, those >90% in *cyan*, and the 19 aa loop in RAC1B is shown in *red*. Conservation of unique RAC1B region is shown in the *red box*. *C*, box and whisker plots for expression of *RAC1-201* and *RAC1-202* transcripts in six different tissues are shown. The RAC1 isoform is in *blue* and the RAC1B isoform is in *orange*. Number of samples within each BioProject are labeled for each sample. Values are shown for transcript per million (TPM) normalization. *D*, proteins encoded by spliced transcripts from the human *RAP1GDS1* gene are shown. The main isoforms are SmgGDS-607/SmgGDS-608, which contain 13 ARM domains A-M, and SmgGDS-558/SmgGDS-559, which lack ARM domain *C*. *E*, evolution of 425 SmgGDS sequences identified on the models of SmgGDS. Amino acids conserved 100% are shown in *blue*, those >90% in *cyan*, and the ARM *C* domain in *red*. Conservation of unique SmgGDS-607 ARM *C* is shown in the *red box*. *F*, box and whisker plots for isoforms of *RAP1GDS1* (SmgGDS) using esophagus BioProject PRJNA626361. The unique *RAP1GDS1* transcript and resulting SmgGDS protein are shown below each bar. Isoforms for SmgGDS-608/SmgGDS-607 are colored in *red*, isoforms for the shorter version SmgGDS-559/SmgGDS-558 in *cyan*, and all others labeled in *black*.

Both RAC1 and RAC1B are able to activate the NF-kB pathway (2, 6) and facilitate the production of reactive oxygen species (7, 8). However, it has been reported that unlike RAC1, RAC1B is not able to bind to RHOGDI, cannot induce lamellipodia formation, and does not signal to p21 activated kinase (PAK) or Jun N-terminal kinase (6). The unique exon present in RAC1B also causes it to have accelerated GDP/GTP exchange, which results in predominantly GTP-bound and active RAC1B within cells (3, 9).

RAC1 and RAC1B are highly expressed in certain types of tumors and can promote cancer progression (4, 9, 10, 11, 12, 13, 14). RAC1B expression was identified predominantly in breast (4, 9, 13, 15), lung (10, 11, 12, 14), and colorectal (4, 15, 16) cancers. Both splice variants were found to promote KRAS-induced lung cancer (11, 17, 18) and the epithelial-mesenchymal transition (12, 18). Interestingly, studies of breast and pancreatic cancers revealed different effects of RAC1 and RAC1B on transforming growth factor (TGF)-β1-induced cell migration (8, 19, 20), where RAC1 promoted and RAC1B inhibited TGF-β1-induced cell motility. In the brain, Rho GTPases, including RAC1, are crucial for the formation, organization, and maturation of neuronal dendrites (21, 22). Misregulation of RAC1, causing alterations in neuronal cytoskeletal organization, has been implicated in neurodegenerative diseases, including Alzheimer’s disease (21). RAC1 splicing has been found to be altered in brains with Alzheimer’s disease and RAC1B expression in some neuronal populations in Alzheimer’s disease has been linked to increased neurofibrillary tangles and membrane dysfunction (21). Misregulation of RAC1 has also been implicated in cardiovascular disease (18), though potential roles of RAC1B in the cardiovascular system have not yet been defined.

Most small GTPases, including RAC1 and RAC1B, are posttranslationally modified by prenylation to facilitate their association with membranes, where small GTPases participate in many signaling pathways. A farnesyl or geranylgeranyl isoprenoid group is added to the cysteine in the C-terminal CAAX motif by farnesyltransferase or geranylgeranyltransferase-I, respectively (23, 24). The specific isoprenoid modification depends on the last amino acid in the CAAX motif and both RAC1 and RAC1B become geranylgeranylated (25). Blocking the prenylation of small GTPases is an attractive therapeutic strategy but farnesyltransferase and GGTase-I inhibitors have proven to be limited in their clinical effectiveness (23, 26), highlighting the need for further investigation of the mechanisms controlling the prenylation pathway.

The chaperone protein SmgGDS has been shown to play a role in regulating the prenylation and trafficking of small GTPases in the Ras and Rho families (27, 28, 29, 30, 31). SmgGDS binds to many different small GTPases that contain a C-terminal polybasic region (PBR) but it is reported to be a guanine nucleotide exchange factor (GEF) only for RHOA and RHOC (32). SmgGDS is encoded by the *RAP1GDS1* gene, which generates 21 different spliced transcripts, and twelve of these alternatively spliced transcripts are reported to generate proteins (Table 1). The best characterized isoforms are the longer SmgGDS-607 isoform and a shorter SmgGDS-558 isoform (27). SmgGDS-558 binds only prenylated small GTPases and helps traffic them throughout the cell (27). In contrast, SmgGDS-607 binds only preprenylated small GTPases and regulates their entry into the prenylation pathway (27). SmgGDS-607 may promote prenylation by delivering preprenylated small GTPases to prenyltransferases (29, 31, 33, 34) but SmgGDS-607 can also suppress prenylation by retaining preprenylated small GTPases and not releasing them to the prenyltransferase (27, 31, 33, 35).

Both SmgGDS-607 and SmgGDS-558 are nucleocytoplasmic shuttling proteins, possessing a nuclear export signal sequence at the N terminus (36, 37). We previously reported similarities between SmgGDS and the nuclear import protein karyopherin-α (36). Both SmgGDS and karyopherin-α contain the WXXXN motif, which is a motif reported to interact with the nuclear localization signal (NLS) in proteins that are transported into the nucleus (36, 38). We showed that RAC1 has a canonical NLS sequence (K(K/R)X(K/R)) in its PBR (36, 39) and that the presence of this sequence promotes nuclear accumulation of RAC1 with SmgGDS (36). RAC1B has the same NLS found in RAC1 and RAC1B has been shown to enter the nucleus (7, 40, 41, 42). However, the interactions for RAC1 and RAC1B with the WXXXN motif in SmgGDS, and potential differences in RAC1 and RAC1B nuclear localization, have not been tested previously.

The tumor suppressor protein DIRAS1 is a Ras family member that binds SmgGDS and diminishes SmgGDS interactions with some small GTPases (33, 43). Like other small GTPases, DIRAS1 has a PBR and is posttranslationally modified by prenylation. DIRAS1 binds with high affinity to both forms of SmgGDS, inhibiting the binding of RHOA, KRAS, and RAP1A to SmgGDS (33, 43). The effects of DIRAS1 on the binding of RAC1 or RAC1B to SmgGDS and its effects on the prenylation of RAC1, RAC1B, or any other small GTPase have not been reported.

Here, we investigate RAC1 and RAC1B to better understand their different expression in human tissues, as well as their differing interactions with SmgGDS and how these interactions affect their prenylation and localization in cells. We identify specific residues in SmgGDS important for RAC1 and RAC1B binding and demonstrate that RAC1B displays enhanced binding to both SmgGDS-607 and SmgGDS-558. Based on the differences in binding to SmgGDS, we predicted that RAC1 and RAC1B isoforms may be prenylated differently, despite their identical PBRs and CAAX motifs. Indeed, we found that RAC1B is only minimally prenylated compared to RAC1 and also accumulates more in the nucleus. Preventing prenylation of RAC1, thus causing it to more resemble RAC1B, increases the nuclear localization of RAC1. In addition, we demonstrate that the expression of the tumor suppressor DIRAS1 decreases the interaction of RAC1 and RAC1B with SmgGDS and decreases their prenylation. Finally, we show that nonprenylated forms of RAC1 and RAC1B are GTP-bound, indicating that RAC1 and RAC1B might actively signal before they are prenylated. Together, these results further illuminate differences in RAC1 and RAC1B interactions, prenylation, and localization, to better define their signaling differences.

## Results

### RAC1 and SmgGDS isoform evolution and expression

The human *RAC1* gene encodes six different transcripts but only two of these transcripts generate stable proteins; RAC1 is translated from the *RAC1-201* transcript and RAC1B is translated from the *RAC1-202* transcript (Table 1 and Fig. 1*A*). RAC1 and RAC1B share similar sequences and structure, except for a loop formed by the 19 additional amino acids that are present only in RAC1B (Fig. 1*A*, and highlighted in red in Fig. 1*B*). Comparing hundreds of sequences encoded by *RAC1* throughout vertebrate evolution, a high conservation of surface-exposed amino acids can be found (Fig. 1*B*). Highly conserved residues in the RAC1B loop (red box, Fig. 1*B*) indicate the importance of splicing that generates the RAC1B protein throughout vertebrate evolution. Human RNA-seq followed by isoform level bioinformatics can resolve the splicing differences within diverse tissues. Analysis of samples from six human tissues indicates different ratios of transcripts encoding RAC1 and RAC1B in different tissues (Fig. 1*C*). We found similar expression of transcripts for RAC1 and RAC1B in esophagus but much greater expression of transcripts for RAC1 than RAC1B in blood and other tissues (Fig. 1*C*). These findings indicate that splicing can generate relatively high amounts of RAC1B in specific tissues (such as the esophagus), where RAC1B might provide unique functions that differ from those of RAC1.

The human *RAP1GDS1* gene encodes multiple transcripts and twelve of these transcripts are reported to generate stable proteins (Table 1 and Fig. 1*D*). The SmgGDS-607 protein is translated from the *RAP1GDS1-205* transcript and is recognized as the canonical sequence. SmgGDS-607 is composed of 13 armadillo (ARM) domains labeled A-M (Fig. 1*D*) (27, 44, 45). SmgGDS-558 is identical to SmgGDS-607 except for the absence of ARM C (Table 1 and Fig. 1*D*). One extra amino acid can be found at the initiator methionine in both the SmgGDS-608 and SmgGDS-559 proteins, compared to the SmgGDS-607 and SmgGDS-558 proteins, respectively (Table 1 and Fig. 1*D*). Several smaller isoforms for SmgGDS have been identified but little is known about their functions (Table 1 and Fig. 1*D*). A comparison of 425 SmgGDS sequences throughout vertebrate evolution revealed a high conservation of surface-exposed amino acids in SmgGDS-607/608, most notably in the binding groove that is reported to bind small GTPases (Fig. 1*E*) (32, 44, 45). Highly conserved residues are also present in ARM C (red box, Fig. 1*E*), which is the structural unit that distinguishes SmgGDS-607/608 from SmgGDS-558/559. Some of these residues are most likely conserved simply because they maintain the 3D structure of the ARMrepeat but others are probably maintained throughout vertebrate evolution because of interactions with conserved binding partners.

As expected, we found significant expression of transcripts that encode the major isoforms SmgGDS-607/608 and SmgGDS-558/559 in tissues (Fig. 1*F*). Unexpectedly, we also found expression of transcripts encoding SmgGDS isoforms that have not yet been characterized (Fig. 1*F*). Most notably, there is relatively high expression of the *RAP1GDS1-220* transcript that encodes the shortest SmgGDS isoform (101 amino acids) (Fig. 1*F*). Nothing is known about the functions of this 101 residue SmgGDS isoform, which consists only of ARM D followed by a C-terminal extension (Fig. 1*D*).

### Identification of a critical lysine in SmgGDS-558 and SmgGDS-607 that is required for binding of RAC1 and RAC1B but not RHOA

**Figure 2:**
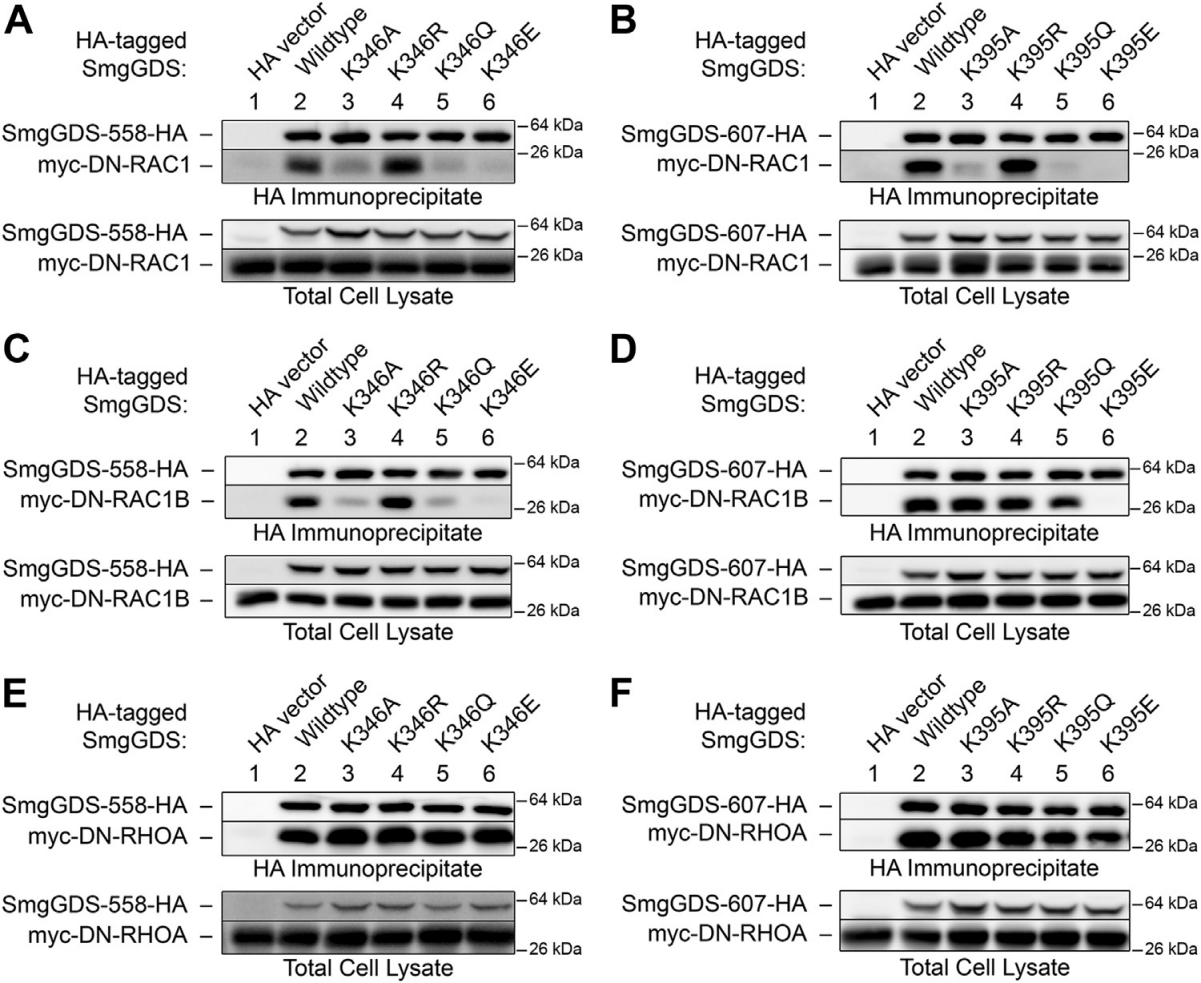
**The charges of K346 and K395 in SmgGDS-558 and SmgGDS-607, respectively, are important for binding of RAC1 and RAC1B but not RHOA.** HEK293T cells were transfected with cDNAs encoding the HA vector, WT or K346 mutant SmgGDS-558-HA (*A*, *C*, *E*), or WT or K395 mutant SmgGDS-607-HA (*B*, *D*, *F*). In addition, the cells were cotransfected with myc-tagged RAC1 (*A* and *B*), RAC1B (*C* and *D*), or RHOA (*E* and *F*) in the dominant negative (DN) form. After 24 h, the cells were lysed and a portion of each lysate was saved for the total cell lysate. The remaining lysate was immunoprecipitated with the HA antibody, followed by ECL immunoblotting using HA and myc antibodies. Results are representative of three independent experiments. Densitometry of the proteins detected in the immunoblots is shown in Fig. S1. The DN form of each small GTPase was used to eliminate potential changes in binding to SmgGDS caused by guanine nucleotide exchange induced by SmgGDS, as previously described (27). ECL, enhanced chemiluminescence.

Small GTPases bind to SmgGDS partially through interactions with the conserved binding groove of SmgGDS (Fig. 1*E*) (32, 44). One important residue in the SmgGDS binding groove, identified originally for its significance in promoting GEF activity for RHOA, is K395 in SmgGDS-607, which corresponds to K346 in SmgGDS-558 (32). We found that mutation of K346 in SmgGDS-558 or K395 in SmgGDS-607 alters the binding of RAC1 and RAC1B, but not RHOA, to SmgGDS (Figs. 2 and S1). Neutralizing the charge of K346/K395 with a mutation to alanine or glutamine decreases the binding of RAC1 to both isoforms of SmgGDS (Fig. 2, *A* and *B*, lanes 3 and 5, and Fig. S1, *A* and *B*). Similarly, mutation of K346 to alanine or glutamine decreases the binding of RAC1B to SmgGDS-558 (Fig. 2*C*, lanes 3 and 5, and Fig. S1*C*). Surprisingly, mutation of K395 in SmgGDS-607 to alanine does not affect binding of RAC1B (Fig. 2*D*, lane 3, and Fig. S1*D*) and mutation of K395 to glutamine only moderately decreases binding of RAC1B (Fig. 2*D*, lane 5, and Fig. S1*D*). Reversing the charge of K346/K395 with a mutation to glutamic acid abolishes the binding of RAC1 and RAC1B to both isoforms of SmgGDS (Fig. 2, *A*–*D*, lane 6, and Fig. S1, *A–D*). While these residues are important for the binding of RAC1 and RAC1B to SmgGDS, RHOA binding is not affected by mutation of K346/K395 (Figs. 2, *E* and *F* and S1, *E* and *F*). Therefore, K346 and K395 in SmgGDS-558 and SmgGDS-607, respectively, are important for the binding of RAC1 and RAC1B to SmgGDS but are not required for binding of all small GTPases.

### A WXXXN motif in SmgGDS facilitates binding to NLS-containing small GTPases, such as RAC1 and RAC1B

**Figure 3:**
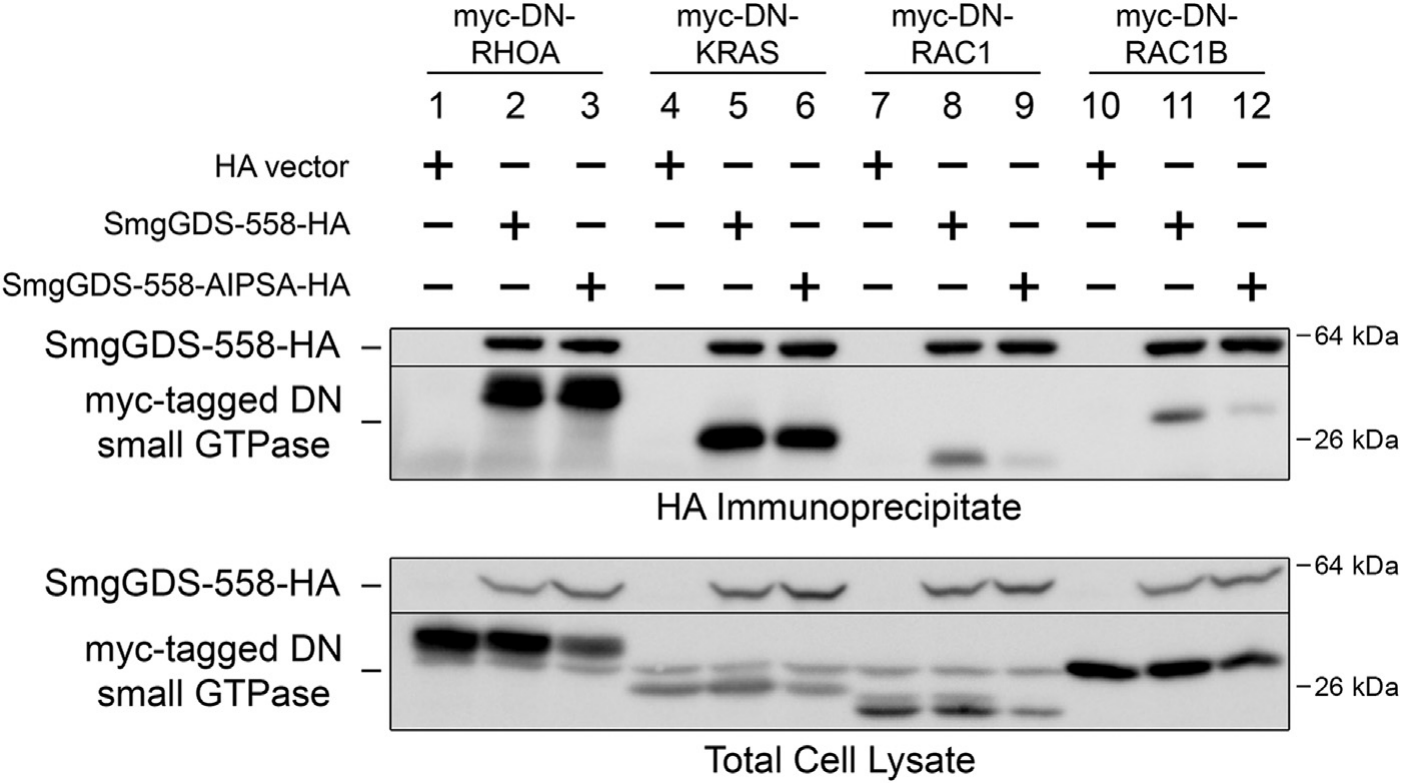
**The *WXXXN* motif in SmgGDS is important for binding of RAC1 and RAC1B but not RHOA or KRAS.** HEK293T cells were transfected with HA vector, SmgGDS-558, or SmgGDS-558-AIPSA-HA, along with the indicated myc-tagged DN small GTPase. After 24 h, the cells were lysed and a portion of each lysate was saved for the total cell lysate. The remaining lysate was immunoprecipitated with the HA antibody, followed by ECL immunoblotting using HA and myc antibodies. Results are representative of three independent experiments. Densitometry of the proteins detected in the immunoblots is shown in Fig. S2. DN, dominant negative; ECL, enhanced chemiluminescence.

SmgGDS is known to bind to small GTPases that contain a C-terminal PBR, which interact with an electronegative patch in SmgGDS (32, 43, 44, 45). We previously reported that the PBR of RAC1 contains an NLS that promotes nuclear accumulation of RAC1/SmgGDS complexes (36). The PBRs of RAC1 and RAC1B are identical, with both containing this NLS. SmgGDS has one WXXXN motif (WIPSN), which is located at amino acids 275–279 in SmgGDS-558 and 324–328 in SmgGDS-607. We hypothesized that the binding of SmgGDS to NLS-containing proteins is similar to that of karyopherin-α, which has three WXXXN motifs at the major NLS binding site and two WXXXN motifs at the minor NLS-binding site (36, 38). To determine the importance of this sequence in SmgGDS-558 for binding to small GTPases containing a NLS in their PBR, we mutated the tryptophan and asparagine in the WIPSN sequence to alanine to create an AIPSA mutant. We then examined the coimmunoprecipitation of the SmgGDS-558-AIPSA mutant with two small GTPases whose PBRs do not contain a NLS, RHOA and KRAS, and with two small GTPases whose PBRs do contain a NLS, RAC1 and RAC1B (Figs. 3 and S2). Mutating the WIPSN sequence to AIPSA in SmgGDS-558 did not alter the interaction with RhoA (Fig. 3, lanes 2 and 3, and Fig. S2*A*) and only minimally affected the interaction with KRAS (Fig. 3, lanes 5 and 6, and Fig. S2*B*), indicating that the WXXXN motif in SmgGDS is not required for these small GTPases. In contrast, much less RAC1 or RAC1B coprecipitated with SmgGDS-558-AIPSA (Fig. 3, lanes 9 and 12, and Fig. S2, *C* and *D*) than with WT SmgGDS-558 (Fig. 3, lanes 8 and 11, and Fig. S2, *C* and *D*). These results are consistent with the WXXXN motif in SmgGDS being required for binding to NLS-containing small GTPases, such as RAC1 and RAC1B.

### Both isoforms of SmgGDS associate more with RAC1B than with RAC1

**Figure 4:**
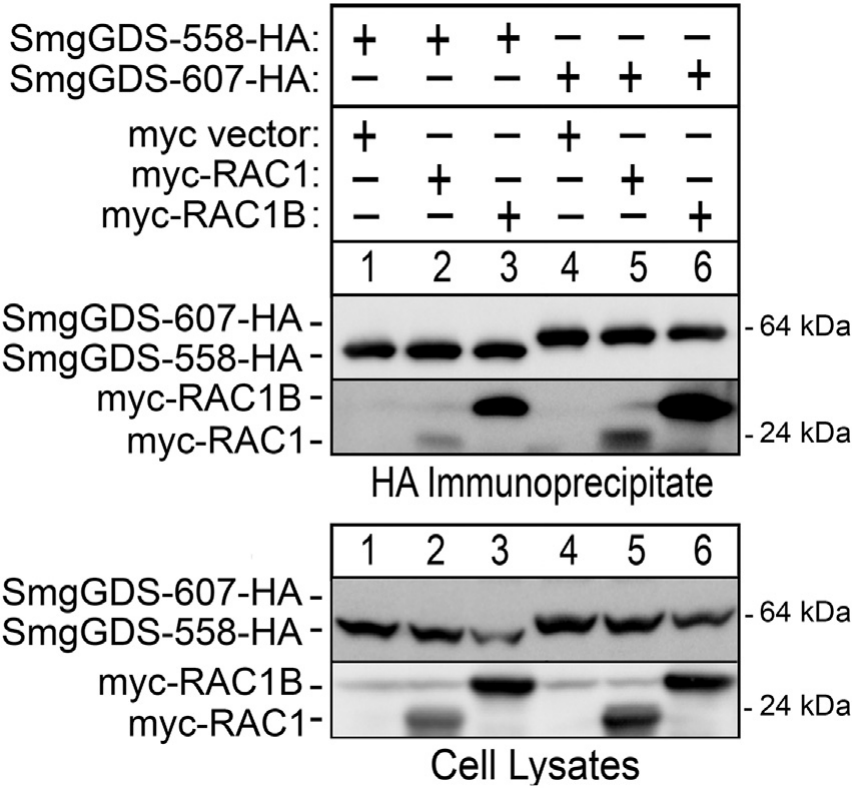
**Compared to RAC1, RAC1B forms a more stable complex with both isoforms of SmgGDS.** HEK293T cells were transfected with SmgGDS-558 or SmgGDS-607, plus myc vector, myc-RAC1, or myc-RAC1B. Twenty-four hours after transfection, the cells were lysed and an aliquot of lysate was retained for total cell lysate immunoblotting. The remaining lysate was immunoprecipitated with HA antibody, and the immunoprecipitations and total cell lysates were immunoblotted using HA and myc antibodies. Results are representative of three independent experiments.

RAC1 and RAC1B possess identical PBRs, but RAC1B is reported to display enhanced binding to some Rho GTPase regulators, including SmgGDS (40). To assess whether RAC1B differs in its binding to SmgGDS-558 compared to SmgGDS-607, we compared the ability of SmgGDS splice variants to coprecipitate RAC1 and RAC1B. WT RAC1 associates with both SmgGDS-607 and SmgGDS-558 (Fig. 4, lanes 2 and 5). Compared to RAC1, we found that RAC1B associates more with both isoforms of SmgGDS (Fig. 4, lanes 3 and 6). Additionally, both RAC1 and RAC1B coprecipitate more with SmgGDS-607, compared to SmgGDS-558 (Fig. 4, lanes 2 and 5, and lanes 3 and 6).

**Figure 5:**
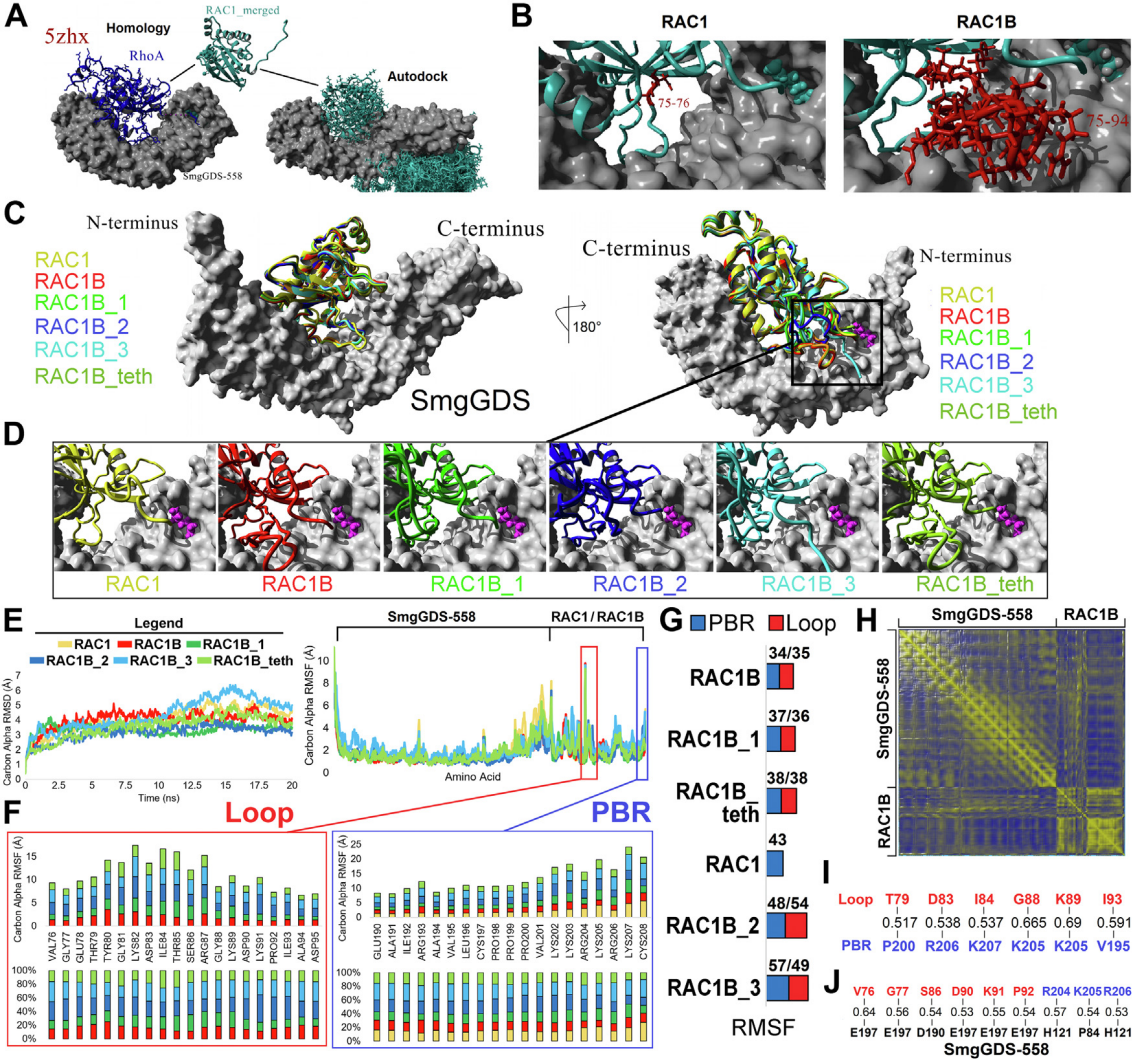
**Modeled interaction of RAC1 or RAC1B with SmgGDS-558.***A*, overlapping modeling approaches used for RAC1-SmgGDS interactions. *B*, the initial binding of RAC1 places the RAC1B loop (*red*) within space for SmgGDS (*gray*) interaction. *C*, predicted interaction of RAC1 (*yellow*) or RAC1B (*red*) with SmgGDS-558 (*gray*) shown in two orientations separated by a 180-degree rotation of the *y*-axis. Four variant conformations of RAC1B (*light green, blue, cyan, dark green*) were generated by sampling, to assess how the RAC1B insert and PBR affect each other’s orientation. The RAC1B_1, RAC1B_2, and RAC1B_3 models depict how different configurations of the insert region affect the position of the C-terminal PBR. The RAC1B_teth model shows the configuration of the insert region when the C terminus is tethered to the RAC1B prenyl group residing in the hydrophobic pocket of SmgGDS-558. *D*, enlarged view of the region boxed in panel *C* for each of the conformations. *E*, molecular dynamic simulations of the global movement as measured by the carbon alpha RMSD in angstroms. Data are shown for 20 nanoseconds (ns). Colors of the line correspond to the initial starting conformations. Amino acid movement of each simulation as measured by the carbon alpha root mean squared fluctuation (RMSF) in angstroms is shown to the *right*. Legend above the plot shows the proteins corresponding to the plotted data. The RAC1B loop (*red*) and polybasic region (PBR, *blue*) are labeled. *F*, details of the movement of the RAC1B loop (*red box*) and polybasic region (PBR, *blue box*). The *top* plot shows the movement of each amino acid stacked for each conformation as colored in panel *C*. The *bottom* plot shows the percent of total movement at the amino acid based on each starting conformation. *G*, the added movement of the PBR (*blue*) and loop (*red*) for each of the starting conformations. They are ranked based on the PBR movement. The *lower* the value the less the region moves. *H*, the dynamics cross correlation matrix of all amino acids of both SmgGDS-558 and RAC1B. The *yellow color* intensity is based on correlation values close to 1 and *blue* close to −1. *I*, the correlations above 0.5 from panel H between residues of the RAC1B loop (*red*) and PBR (*blue*). *J*, the correlations above 0.5 from panel H between residues of the RAC1B loop (*red*) or PBR (*blue*) relative to SmgGDS-558 (*black*). PBR, PBR, polybasic region.

**Figure 6:**
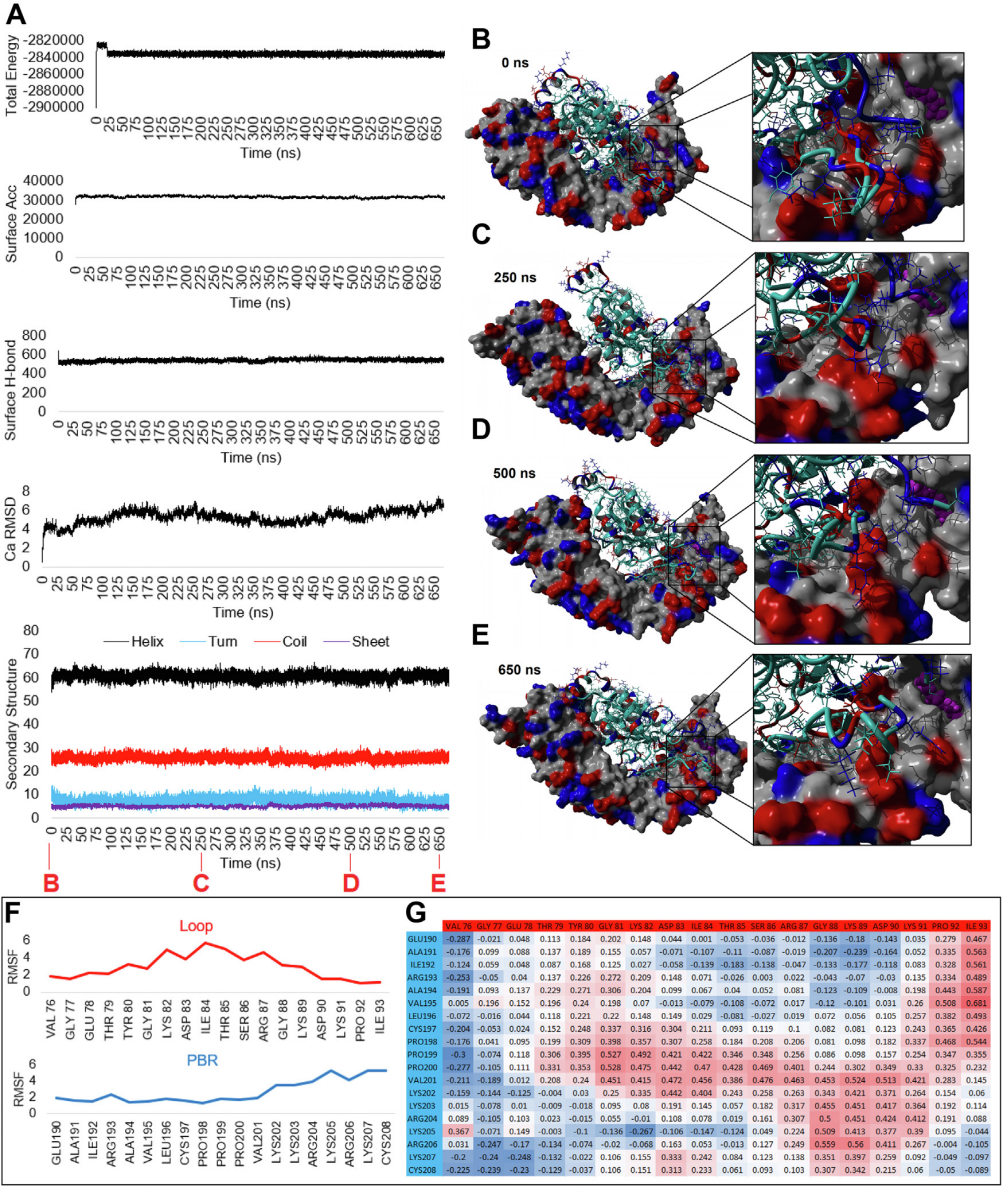
**Simulations of the RAC1B conformation for 650 nanoseconds show stability of the loop region relative to the PBR.***A*, various statistics for the 650 nanosecond (ns) simulation of RAC1B with SmgGDS for 26,000 data points. Shown are the total energy, surface accessible area of all proteins, surface hydrogen bonds with water, Carbon Alpha RMSD, and percent of each secondary structure annotations. All metrics show the equilibrium of the simulation. *B–E*, representative time points shown in *red* on panel *A* for the RAC1B (*cyan*) interaction with SmgGDS shown as a surface plot. The lipidation site is colored *magenta*, polar basic residues in *blue*, and polar acid residues in *red*. Sampled times include 0 ns (*B*), 250 ns (*C*), 500 ns (*D*), and 650 ns (*E*). *F*, average movement of RAC1B loop (*red*) or polybasic region (PBR, *blue*) amino acids throughout the entire long simulation. *G*, dynamic cross-correlation analysis of loop (*Top*) relative to PBR (*Side*) amino acids. The numbers are colored on a heatmap of *red* being highly correlated and *blue* being negatively correlated. PBR, PBR, polybasic region.

To examine how the additional 19 amino acids in RAC1B contribute to the greater association of RAC1B with SmgGDS, we performed modeling, loop sampling, and molecular dynamics (MD) simulations (Fig. 5). Few structures have been solved for SmgGDS proteins relative to small GTPases such as RAC1; however, the recent RHOA-SmgGDS model (44, 45) gives an idea of potential interactions. Two independent modeling approaches (autodock and RAC1 replacement within 5zhx) yielded similar binding sites for RAC1 to SmgGDS (Fig. 5*A*). Following sampling and energy minimization for the docking data, RAC1 binding was used to further model several conformations of the 19 amino acids of RAC1B relative to SmgGDS (Fig. 5*B*). Comparing models of RAC1 (yellow) to RAC1B (red), we found that residues in the insert of RAC1B likely form a loop that is in the perfect position to interact with residues in SmgGDS, which could enhance SmgGDS binding of RAC1B relative to RAC1 (Fig. 5, *C* and *D*). Modeling of multiple conformations of the RAC1B insert and PBR were performed, followed by assessment of binding using short MD simulation to quantify each amino acid’s interactions (Fig. 5, *C* and *D*). This also allowed for multiple assessments of the interactions between the insert and the C-terminal PBR (Fig. 5*D*), yielding an increased sampling of space of the RAC1B loop relative to longer more computationally risky MD simulations that can get stuck in local minimum states. Each conformation was simulated for 20 nanoseconds (ns) and showed relative global stability over time (Fig. 5*E*). The total movement of each amino acid over the 20 ns simulation showed similar trajectories in each simulation (Fig. 5*F*). Movement within the loop region in RAC1B indicates stability of multiple residues in each starting conformation, yet several residues show stabilized movement in the RAC1B lowest energy minimized conformation (red box, Fig. 5*F*). Similarly, the PBR region of RAC1B shows stability of amino acids 190-201, with additional stability of amino acids 202-208 relative even to RAC1 (blue box, Fig. 5*F*). RAC1B was the most stable (lowest overall total RMSF movement) throughout the simulation for both the insert and PBR, with more stability of the PBR when the loop was present (Fig. 5*G*). The PBR conformation was identical between the RAC1 and RAC1B initial models and thus the lower movement of the RAC1B PBR suggests that the loop can potentially synergize with the PBR for SmgGDS interaction. Similar results were obtained by longer simulations of the top RAC1B conformation up to 650 ns, showing stability of the loop region relative to the PBR over increased sampling of the movement (Fig. 6).

We applied dynamics cross correlation matrix analysis of the RAC1B conformation to determine how each amino acid correlates intramolecularly and intermolecularly with every other amino acid in the simulation (Fig. 5*H*). There is a correlation of several amino acids within the unique loop of RAC1B and the shared PBR including T79-P200, D83-R206, I84-K207, G88-K205, K89-K205, and I93-V195 (Fig. 5*I*). Several of the amino acids in the RAC1B loop are also correlated with SmgGDS, including RAC1B V76 to SmgGDS-558 E197 (V76-E197), G77-E197, S86-D190, D90-E197, K91-E197, and P92-E197 (Fig. 5*J*). Three residues of the RAC1B PBR are correlated with SmgGDS, including R204-H121, K205-P84, R206-H121 (Fig. 5*J*). Residues K205 and R206 are the only two residues of RAC1B to correlate with both the loop region and with amino acids in SmgGDS. This finding suggests that not only does the insert region of RAC1B provide additional contacts to increase the stability of RAC1B with SmgGDS but it can also stabilize the PBR of RAC1B onto SmgGDS. This further explains the greater association of RAC1B with SmgGDS than the association of RAC1 with SmgGDS.

### RAC1B exhibits less prenylation and more nuclear localization than RAC1

**Figure 7:**
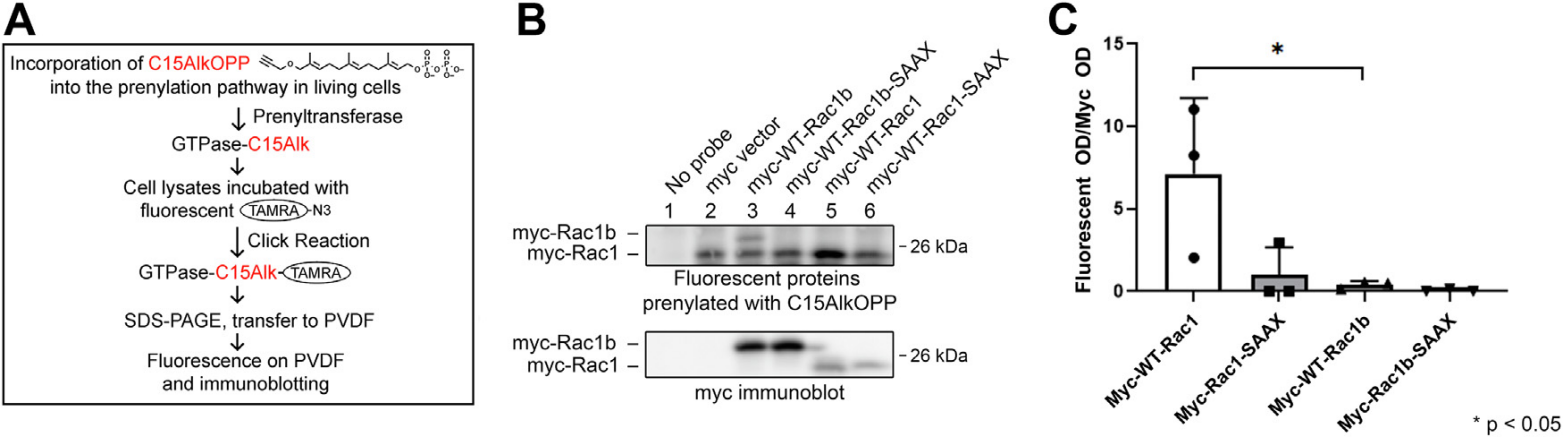
**Compared to RAC1, RAC1B is only minimally prenylated in HEK293T cells.***A*, diagram depicting the use of the C15AlkOPP isoprenoid probe for measuring prenylation of expressed small GTPases. HEK293T cells were transfected with myc-tagged RAC1 or RAC1B and theC15AlkOPP probe was added 90 min after transfection. The probe was allowed to incorporate into the prenylation pathway for 18 h. Cells were lysed and labeled proteins were subjected to click reaction with TAMRA PEG_3-_N_3_. TAMRA-labeled proteins were run on SDS-PAGE gels, transferred to PVDF, and analyzed for fluorescence and for total myc-tagged protein expression by immunoblotting with myc antibody. *B*, fluorescence analysis and myc immunoblotting was performed on lysates of HEK293T cells. Cells not incubated with the C15AlkOPP probe were used as a negative control (*lane 1*) and cells expressing only myc vector were used to account for background labeling of endogenous small GTPases (*lane 2*). Fluorescence and total myc-tagged protein are shown for cells expressing myc-tagged WT RAC1B (*lane 3*), nonprenylated RAC1B-SAAX (*lane 4*), WT RAC1 (*lane 5*), or nonprenylated RAC1-SAAX (*lane 6*). *C*, optical densities of fluorescent protein were normalized to optical densities of myc-tagged protein and are shown as mean ± SD (n = 3). Statistical significance was determined using one-way ANOVA followed by Tukey’s multiple comparisons posthoc test (∗*p* < 0.05).

SmgGDS is a critical regulator of small GTPase prenylation and trafficking; SmgGDS-607 binds only preprenylated small GTPases and SmgGDS-558 binds only prenylated small GTPases (27, 31). Since RAC1B displays greater association with both isoforms of SmgGDS (Figs. 4 and 5), we hypothesized that this might cause differences in prenylation of RAC1 and RAC1B. To examine protein prenylation, we utilized click chemistry with an isoprenoid analog to measure the amounts of prenylated RAC1 and RAC1B. After transfecting HEK293T cells with myc-tagged RAC1 or RAC1B, cells were incubated with the isoprenoid analog probe containing a reactive alkyne group (Fig. 7*A*). The probe passively enters the cells and is utilized by prenyltransferases to prenylate CAAX-containing proteins. After cell lysis, we incubated the lysates with fluorescent tetramethylrhodamine (TAMRA)-N_3_ and conducted the click reaction (Fig. 7*A*). Proteins were resolved using an SDS-PAGE gel and transferred to polyvinylidene difluoride (PVDF). We then imaged the PVDF to detect fluorescently labeled, prenylated proteins, followed by immunoblotting using myc antibody to detect expression of the myc-tagged RAC1 and RAC1B. (Fig. 7*A*).

We detected background labeling of endogenous small GTPases that were prenylated in cells transfected with the myc vector control (Fig. 7*B*, lane 2). Only background labeling of endogenous small GTPases occurred in cells transfected with the myc-tagged RAC1–SAAX and RAC1B–SAAX mutants, which have serine replacing the cysteine in the CAAX motif, preventing prenylation and acting as a negative control for the myc-tagged WT proteins (Fig. 7*B*, lanes 4 and 6). There is significant prenylation of myc-tagged RAC1 in the cells, as indicated by the greater fluorescent signal in the lysates from cells expressing this protein (Fig. 7*B*, lane 5). In contrast, myc-tagged RAC1B is minimally prenylated, as indicated by the weak fluorescent signal corresponding to myc-RAC1B, which migrates more slowly than RAC1 due to the greater molecular weight of RAC1B (Fig. 7*B*, lane 3).

**Figure 8:**
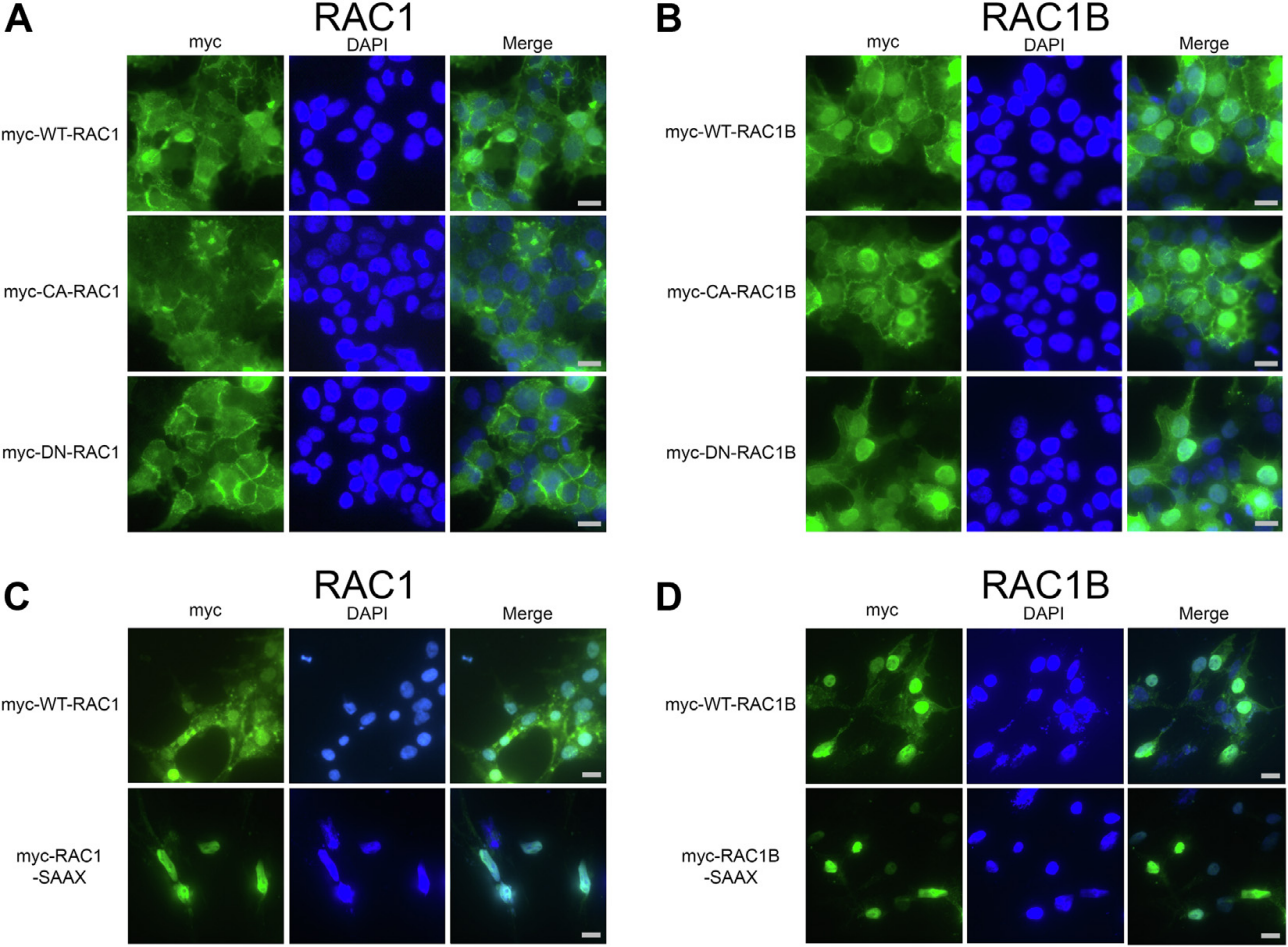
**Compared to RAC1, RAC1B is more nuclear, and the SAAX mutation of RAC1 and RAC1B increases nuclear localization.** HEK293T cells were transfected with WT, constitutively active (CA), or dominant negative (DN) (*A*) RAC1 or (*B*) RAC1B and analyzed by immunofluorescence to determine subcellular localization. HEK293T cells were also transfected with WT or nonprenylated SAAX mutants of (*C*) RAC1 or (*D*) RAC1B. The *gray* scale bars represent 10 μm. Results are representative of three independent experiments.

Prenylated RAC1 often localizes at the cell membrane or is sequestered in the cytoplasm when bound to RHOGDI (46). However, RAC1 also contains a NLS in its PBR and can be shuttled into the nucleus (36). We found that WT, constitutively active, and dominant negative RAC1 predominantly localize to the membrane and the cytoplasm, with some nuclear localization (Fig. 8*A*). RAC1B contains the same NLS as RAC1, and RAC1B has been reported to enter the nucleus (7, 40, 41, 42). Because RAC1B is minimally prenylated, we hypothesized that RAC1B would display greater nuclear localization than RAC1. Consistent with this hypothesis, we detected greater nuclear localization of WT, constitutively active, and dominant negative RAC1B (Fig. 8*B*), than the nuclear localization of RAC1 (Fig. 8*A*). Mutating the RAC1 CAAX motif to SAAX prevents prenylation of RAC1, causing RAC1 to act similarly to RAC1B and accumulate more in the nucleus (Fig. 8*C*). The nuclear localization of RAC1B is also slightly enhanced by mutating the RAC1B CAAX motif to SAAX (Fig. 8*D*). Together, these findings indicate that the minimally prenylated state of RAC1B facilitates its nuclear localization and that decreasing RAC1 prenylation causes it to more closely adopt RAC1B localization patterns and accumulate in the nucleus.

### Expression of DIRAS1 decreases RAC1 and RAC1B prenylation

**Figure 9:**
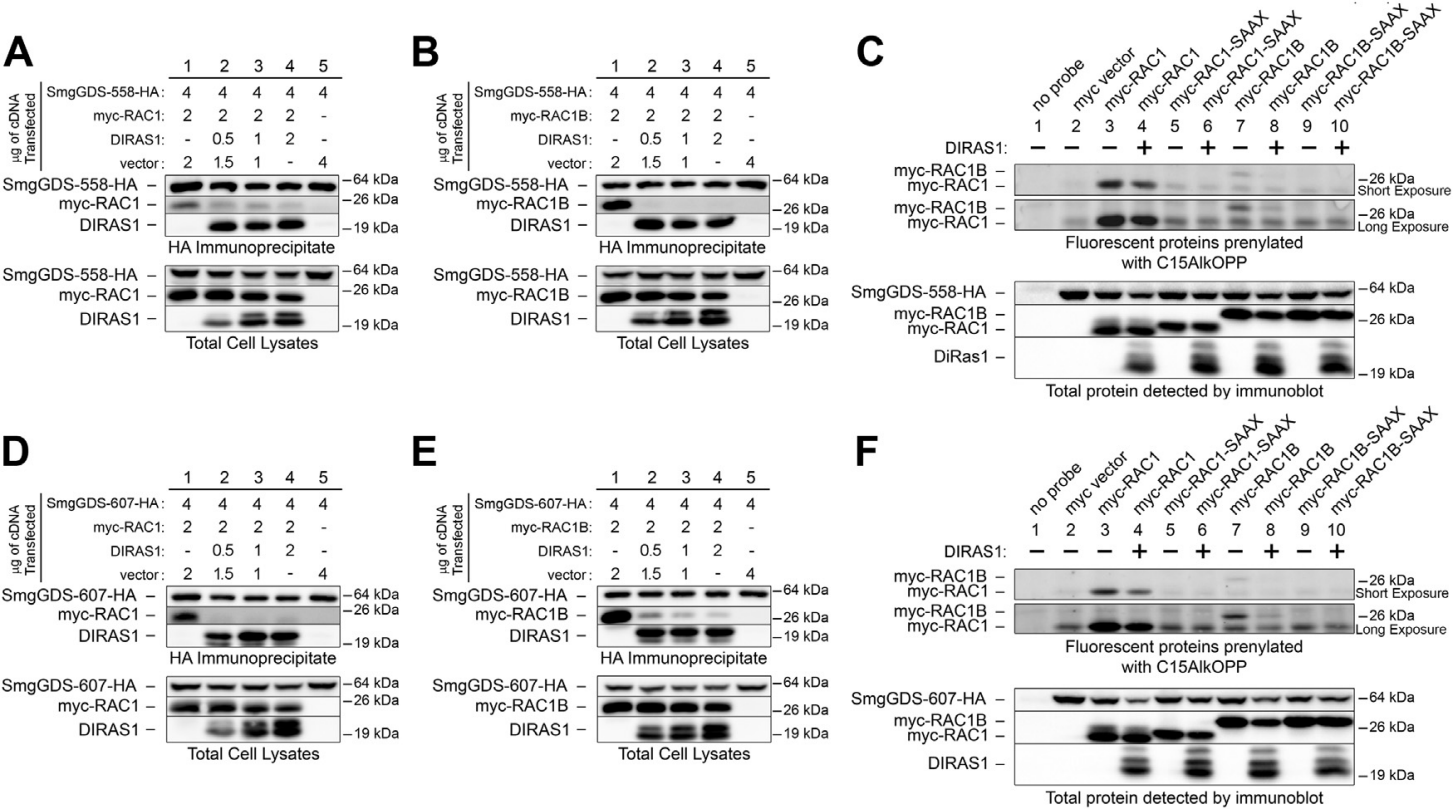
**DIRAS1 inhibits RAC1 and RAC1B binding to SmgGDS and reduces their prenylation.** HEK293T cells were cotransfected with cDNAs encoding SmgGDS-558-HA and myc-tagged (*A*) RAC1 or (*B*) RAC1B along with increasing amounts of DIRAS1 cDNA and/or empty vector, such that all cells were transfected with equal amounts of total cDNA. HEK293T cells cotransfected with SmgGDS-607-HA and myc-tagged (*D*) RAC1 or (*E*) RAC1B cDNAs were also transfected with increasing amounts of DIRAS1 cDNA and/or empty vector in the same concentrations as in *A* and *B*. After 24 h, the cells were lysed and a portion of each lysate was saved for the total cell lysate. The remaining lysate was immunoprecipitated with the HA antibody, followed by ECL immunoblotting using HA and myc antibodies. To evaluate prenylation of RAC1 and RAC1B in the presence of DIRAS1, cells were transfected with myc-tagged RAC1 or RAC1B plus (*C*) SmgGDS-558 or (*F*) SmgGDS-607, in the presence or absence of cotransfected DIRAS1. Prenylation with the C15AlkOPP probe was measured as described in Figure 7. Results are representative of three independent experiments. Densitometry of the proteins detected in the immunoblots is shown in Fig. S3. ECL, ECL, enhanced chemiluminescence.

The tumor suppressor protein DIRAS1 inhibits binding of RHOA, KRAS, and RAP1A to SmgGDS (43), providing a mechanism that may regulate the interactions of SmgGDS and small GTPases. We examined whether DIRAS1 also regulates SmgGDS interactions with RAC1 and RAC1B. As expected, our results indicate that DIRAS1 similarly affects RAC1 and RAC1B, since we found that DIRAS1 inhibits the interaction of both RAC1 and RAC1B with SmgGDS-607 and SmgGDS-558 (Figs 9, *A*, *B*, *D*, *E*, and S3, *A–D*). We hypothesized that DIRAS1 can suppress the prenylation of RAC1 and RAC1B by inhibiting their association with SmgGDS-607. This hypothesis is based on previous reports that SmgGDS-607 facilitates the entry of some small GTPases, such as RAP1B, into the prenylation pathway (29). Consistent with our hypothesis, RAC1B prenylation is significantly reduced in cells expressing DIRAS1 (Fig. 9, *C* and *F*, compare lanes 7 and 8, long exposure, and Fig. S3, *F* and *H*). To a lesser extent, RAC1 prenylation is also decreased when DIRAS1 is expressed (Fig. 9, *C* and *F*, compare lanes 3 and 4, short exposure, and Fig. S3, *E* and *G*). These findings provide greater insight into the tumor suppressive functions of DIRAS1, supporting the model that DIRAS1 diminishes the ability of small GTPases to enter the prenylation pathway by inhibiting their interaction with SmgGDS-607.

### Nonprenylated RAC1 and RAC1B are GTP-bound

**Figure 10:**
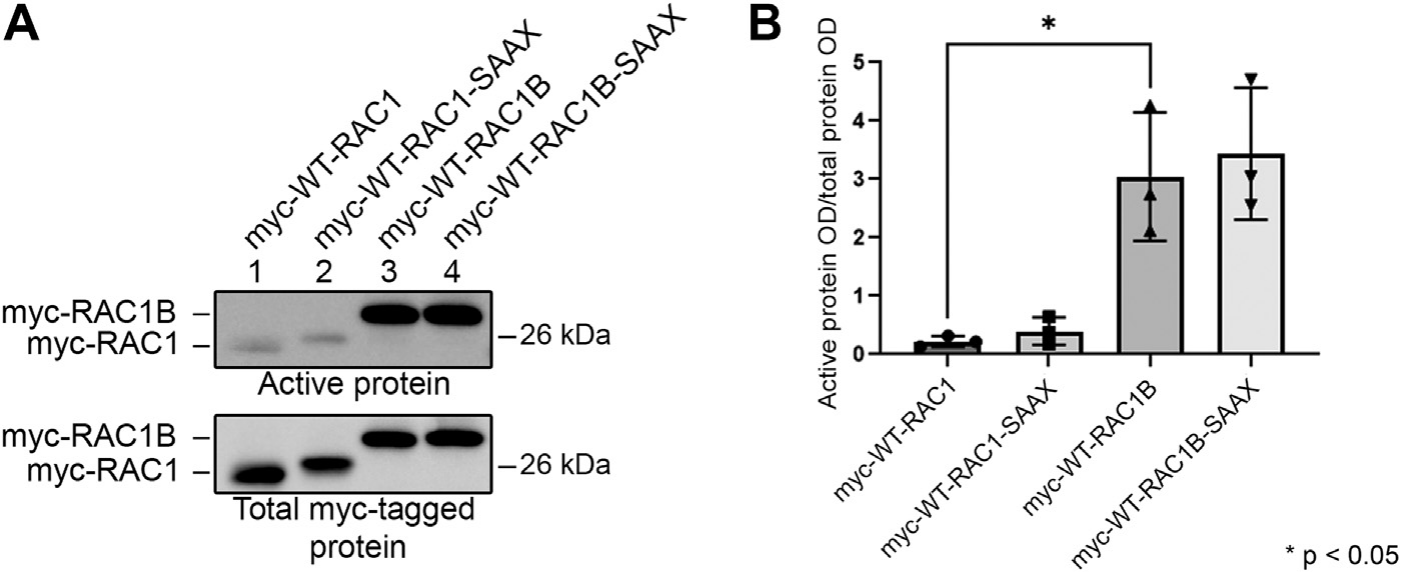
**Nonprenylated SAAX mutants of RAC1 and RAC1B are GTP-bound in HEK293T cells.***A*, HEK293T cells were transfected with myc-tagged WT or SAAX mutant RAC1 or RAC1B, and the GTP-bound small GTPases were detected using a RAC pull down activation assay, followed by immunoblotting with myc antibody. *B*, optical densities of GTP-bound RAC1 or RAC1B were normalized to optical densities of total expressed RAC1 or RAC1B and are shown as mean ± SD (n = 3). Statistical significance was determined using one-way ANOVA followed by Tukey’s multiple comparisons posthoc test (∗*p* < 0.05).

Classically, it has been thought that small GTPases must be prenylated and localize at the cell membrane to be GTP-bound and to participate in signaling cascades. However, more recent studies demonstrate that small GTPases can be GTP-bound without previous prenylation (47, 48, 49). To examine the GTP-bound state of WT and the nonprenylated SAAX mutants of RAC1 and RAC1B, we used assays in which the GTP-bound form of RAC1 and RAC1B were isolated from HEK293T cell lysates by coprecipitation with the RAC-binding domain of PAK1, which recognizes only the GTP-bound form of the small GTPases. This assay indicated significantly greater GTP-binding by RAC1B than RAC1 in these cells (Fig. 10*A*, lanes 1 and 3, and Fig. 10*B*), consistent with reports that RAC1B is the more active isoform (3, 9). Interestingly, the nonprenylated SAAX mutants of both RAC1 and RAC1B are also GTP-bound (Fig. 10*A*, lanes 2 and 4), and in fact, there are similar amounts of GTP-bound WT and SAAX-mutant protein present for both RAC1 and RAC1B. These results indicate that both RAC1 and RAC1B can be activated in cells before they are prenylated.

## Discussion

This study expands our understanding of the differences between RAC1 and RAC1B interactions, prenylation, and localization. Compared to other small GTPases, RAC1 and RAC1B have unique requirements for binding to SmgGDS, at least partially due to the NLS in the PBR shared by both isoforms. However, despite having identical PBRs, we found that RAC1 and RAC1B differ in their binding to SmgGDS, likely contributing to their differences in prenylation and localization. In combination, these findings help explain the reported differences in signaling displayed by RAC1 and RAC1B, which could assist in therapeutic targeting of these splice isoforms either together or individually.

Studies of the binding of small GTPases to SmgGDS often focus on the PBRs of the small GTPases and the conserved binding groove of SmgGDS. In this regard, it is interesting that RAC1 and RAC1B display differences in their stable association with SmgGDS, since these proteins have identical PBRs. Our modeling indicates that the presence of the 19 amino acid insertion in RAC1B facilitates the more stable association of RAC1B with SmgGDS in two ways. Firstly, these additional amino acids extend to form a loop that provides additional contacts to SmgGDS that are not available for RAC1 (Fig. 5, *C* and *D*). Secondly, and somewhat surprisingly, we found that the loop and the PBR of RAC1B likely stabilize each other (Fig. 5*F*). It is expected that more advanced structural analysis in the future will support our model that the RAC1B loop and PBR give additional stability to RAC1B/SmgGDS complexes. Importantly, our current model is consistent with our experimental data showing that SmgGDS coprecipitates more with RAC1B than RAC1.

Previously, several residues within the SmgGDS conserved binding groove were evaluated for their importance in promoting GEF activity of SmgGDS for RHOA and RHOC (32). K395 in SmgGDS-607 and the cognate K346 in SmgGDS-558 are highly conserved and mutation of K395 or K346 was shown to decrease the ability of SmgGDS-607 or SmgGDS-558, respectively, to promote guanine nucleotide exchange by RHOA (32). While K395 in SmgGDS-607 and K346 in SmgGDS-558 are important for activation of RHOA (32), our results indicate that these lysines are not required for binding of RHOA to SmgGDS (Fig. 2, *E* and *F*). In contrast, we found that these residues play a significant role in the binding of RAC1 and RAC1B to SmgGDS (Fig. 2, *A*–*D*).

The reason why K346 in SmgGDS-558 (and the cognate K395 in SmgGDS-607) participate in binding of RAC1 and RAC1B, but not RHOA, is due to structural differences between these small GTPases. The Shimizu laboratory reported that the N-terminal R5 and K6 residues in RHOA form salt bridges with E164 and E162, respectively, in SmgGDS-558, promoting binding of RHOA to SmgGDS-558 (44). RAC1 lacks these N-terminal residues, supporting the conclusion that RAC1 and RHOA have different structural interactions with SmgGDS (44). Interestingly, the Shimizu group identified R337 and K372, but not K346, in SmgGDS-558 as residues that participate in the binding of RHOA (44). Our results are consistent with this report that K346 in SmgGDS-558 is not needed for binding RHOA. However, since this residue promotes SmgGDS GEF activity for RHOA (32), this residue may be needed for SmgGDS to structurally alter the Switch-II region of RHOA to induce nucleotide exchange (44).

These results highlight the ability of SmgGDS to interact with different small GTPases in different ways. SmgGDS-558 interacts with the PBR, prenyl group, and G-domain of RHOA (44, 45). In contrast, we reported that SmgGDS-558 interacts with the PBR and prenyl group, but not the G-domain, of KRAS (50). Our finding that the WXXXN motif in SmgGDS-558 is critically involved in binding RAC1 and RAC1B, but not RHOA or KRAS (Fig. 3), indicates that SmgGDS-558 interacts in a unique way with RAC1 and RAC1B. These different interactions have functional significance. The ability of SmgGDS to be a GEF for RHOA but not for KRAS or RAC1 (32) most likely occurs because SmgGDS engages the G-domain of RHOA but not KRAS or RAC1 (44, 50). It is currently unknown if SmgGDS has GEF activity for RAC1B. Our discovery that SmgGDS interacts with the G domain of RAC1B by engaging the19 amino acid loop near the Switch II region provides a rationale for future studies examining the ability of SmgGDS to modulate the nucleotide-bound state of RAC1B.

We observed intriguing differences in how RAC1 and RAC1B interact with K395 in SmgGDS-607. Neutralizing K395 in SmgGDS-607 by substitution with alanine or glutamine diminishes binding of RAC1 but does not diminish binding of RAC1B. The additional contacts between RAC1B and SmgGDS-607 provided by the 19 amino acid loop in RAC1B (Figs. 1*B* and 5) appear to be enough to overcome the loss of the positive charge at K395 and maintain association with SmgGDS-607. However, the additional contacts provided by the RAC1B loop are apparently not strong enough to surmount the introduction of a negative charge at this site in SmgGDS-607, because binding of RAC1B to SmgGDS-607 is lost when K395 in SmgGDS-607 is replaced with a negatively charged glutamic acid (Fig. 2*D*).

Interestingly, our results indicate that RAC1 and RAC1B do not differ in their responses to disruption of K346 in SmgGDS-558, despite their different responses to mutation of K395 in SmgGDS-607. Both RAC1 and RAC1B exhibit less interaction with SmgGDS-558 when K346 in SmgGDS-558 is neutralized by substituting alanine or glutamine, or when its charge is reversed by substituting glutamic acid. The similarities in binding patterns between RAC1 and RAC1B to SmgGDS-558 are likely due to the presence of the prenyl group. RAC1 and RAC1B will only bind to SmgGDS-558 when they are prenylated and the presence of the highly hydrophobic prenyl group may drive binding or constrain both splice variants in similar ways, which could result in the comparable binding patterns to these SmgGDS-558 K346 mutants. The 19 amino acid loop in RAC1B clearly modulates its interactions with SmgGDS but prenylation of this splice variant also plays a crucial role in binding to SmgGDS-558.

SmgGDS-607 is thought to be a gatekeeper for the prenylation pathway, controlling the entry of preprenylated small GTPases into the prenylation pathway. SmgGDS-607 can promote prenylation by binding preprenylated small GTPases and delivering them to the prenyltransferase (29, 31, 33, 34) but SmgGDS-607 can also suppress prenylation by not releasing preprenylated small GTPases to the prenyltransferase (27, 31, 33, 35). Small GTPases that form very stable complexes with SmgGDS-607 may be less prenylated because they are less likely to be released to the prenyltransferase. The stable association of RAC1B with SmgGDS-607 may cause RAC1B to be less prenylated than RAC1, which forms less stable complexes with SmgGDS-607. The participation of SmgGDS in causing the observed differences in prenylation of RAC1 and RAC1B is strengthened by the fact that these differences in prenylation cannot be attributed to their PBRs and CAAX motifs, since these sequences are identical in RAC1 and RAC1B.

Some small GTPases may utilize SmgGDS-607 to deliver them to the prenyltransferase (29, 31, 33, 34). According to this model, a preprenylated small GTPase that cannot initially bind SmgGDS-607 will have reduced prenylation. This model is supported by reports that the small GTPase RAP1B is not prenylated if it cannot bind SmgGDS-607 due to phosphorylation of the RAP1B PBR (29, 34). Intriguingly, we found that expression of DIRAS1 inhibits binding of RAC1 and RAC1B to SmgGDS and decreases their prenylation. This finding suggests that both RAC1 and RAC1B utilize SmgGDS-607 for delivery to the prenyltransferase. However, additional events not involving SmgGDS most likely regulate prenylation, since we observed that DIRAS1 expression completely inhibited detectable association of RAC1 with SmgGDS-607, and yet RAC1 continued to be significantly prenylated in the cells. DIRAS1 is a well-known tumor suppressor (43) and the ability of DIRAS1 to significantly diminish the prenylation of RAC1B, and to a lesser extent RAC1, provides a new mechanism through which DIRAS1 may inhibit oncogenic signaling by these small GTPases.

We previously hypothesized that the WXXXN motif in SmgGDS is important for binding of NLS-containing proteins, similar to the WXXXN motifs that are used by karyopherin-α as NLS binding sites (36). We observed that this WXXXN motif in SmgGDS is required for binding of RAC1 and RAC1B, whose PBRs contain an NLS, and it is not necessary for binding of KRAS and RHOA, whose PBRs do not contain an NLS (Fig. 3). This observation is consistent with the ability of SmgGDS to shuttle RAC1 isoforms between the nucleus and the cytoplasm (36). We demonstrated that RAC1B localizes more to the nucleus than RAC1 (Fig. 8). The minimal prenylation of RAC1B and the reported lack of interactions with RHOGDI (6) likely allow for RAC1B to more freely shuttle to the nucleus, assisted by its NLS. Regardless of having the same NLS, RAC1 is more likely to be kept at the cell membrane by its prenyl group or to be sequestered in the cytoplasm by RHOGDI. These localization differences may be additionally exacerbated by the greater association of SmgGDS with RAC1B than with RAC1, which may allow for greater shuttling of RAC1B to the nucleus.

Despite classical views that small GTPases must be prenylated before participating in signaling pathways, growing evidence suggests that small GTPases can be activated before being prenylated (47, 48, 49). We report here that nonprenylated RAC1 and RAC1B mutants significantly bind GTP in cells (Fig. 10). This finding has important implications for RAC1B, since our results indicate that a large proportion of RAC1B remains in the preprenylated state in cells. This preprenylated RAC1B may have important signaling roles, in addition to its signaling roles after it is prenylated. The presence of a NLS and the ability of SmgGDS to shuttle RAC1B between the nucleus and the cytoplasm speak to its potential importance in various subcellular compartments. SmgGDS-607 may regulate the localization of preprenylated RAC1B and shuttle it into the nucleus for signaling. Upon receipt of a different signal, SmgGDS-607 may also escort preprenylated RAC1B back to the cytoplasm for prenylation or for other signaling events mediated by pre-prenylated RAC1B in the cytosol. SmgGDS-558 is likely to perform a similar role for prenylated RAC1 and RAC1B. The variable isoform expression levels in different tissues (Fig. 1*C*) is consistent with the differential regulation of RAC1 and RAC1B expression in different cell types (5).

Collectively, our findings indicate that RAC1 and RAC1B differ in their interactions with the chaperone protein SmgGDS, contributing to distinct differences in their prenylation and localization. Our results provide insights into some of the reported differences in RAC1 and RAC1B regulation and function in cells (6). For example, the minimal prenylation of RAC1B might cause it to minimally associate with RHOGDI in cells (6), since RHOGDI binds the prenyl group of small GTPases (51). Additionally, the greater nuclear accumulation of RAC1B might contribute to its reduced ability to promote lamellipodia formation and to signal to PAK or Jun N-terminal kinase (6), since these events generally involve interactions with proteins outside of the nucleus. On a broader scale, our results provide further evidence of the cellular regulation of small GTPase prenylation, at least partially regulated by SmgGDS, as opposed to the constitutive prenylation of small GTPases immediately after translation. Understanding how the prenylation and localization of small GTPases such as RAC1 and RAC1B are regulated will help define new strategies to target small GTPases in various diseases, including cancers and neurologic or cardiovascular disorders.

## Experimental procedures

### Evolution analysis of isoforms

The vertebrate sequences of *RAC1* and *RAP1GDS1* (SmgGDS) were extracted from NCBI ortholog (52), ORF extracted using Transdecoder, and protein sequences aligned using ClustalW (53). The splice sites were identified based on UniProt annotation. Homology modeling, conservation mapping, and image generation were performed for RAC1B (UniProt P63000-2) and SmgGDS 607 (P52306-1) using YASARA modeling (54).

### Tissue expression of isoforms

The RNA-seq datasets of the esophagus (BioProject PRJNA626361), pancreas (PRJNA248621), endometrium (PRJNA384963), liver (PRJNA310012), blood (PRJNA400331), and skin (PRJNA385075) were performed. The read files (fastq) were downloaded using the SRAtoolkit (55) by splitting the paired end reads and processed with Salmon (56) quasi alignment relative to the human Gencode39 transcriptome database (57).

### Cell culture and cDNA transfection

HEK293T cells were obtained from the American Type Culture Collection. HEK293T cells were maintained in Dulbecco's modified Eagle's mediumhigh glucose with L-glutamine media supplemented with sodium pyruvate, 10% heat-inactivated fetal bovine serum, and penicillin/streptomycin.

The cDNAs encoding C-terminal hemagglutinin (HA)-tagged SmgGDS and N-terminal myc-tagged small GTPases were generated as described (27, 36, 43). Site-directed mutagenesis of cDNAs encoding SmgGDS, RAC1, and RAC1B was performed using QuikChange II Site-Directed Mutagenesis Kit (Stratagene) and confirmed by sequencing. All cDNAs were transfected using Lipofectamine 2000 (Life Technologies), according to the manufacturer’s protocol.

### Immunoprecipitation assays

HEK293T cells were transfected with the indicated cDNAs encoding SmgGDS-HA and myc-tagged small GTPases. The cells were lysed in 0.5% Nonidet-P40 with protease and phosphatase inhibitors and centrifuged at 2500*g* for 5 min at 4 °C. An aliquot of cleared lysate was saved for total cell lysate and the remainder was immunoprecipitated using HA-conjugated agarose beads (Sigma). Total cell lysates and immunoprecipitates were analyzed by enhanced chemiluminescence immunoblotting.

### Immunoblotting

Equal numbers of transfected cells were boiled in Laemmli sample buffer for 5 min and subjected to SDS-PAGE. Proteins were transferred to PVDF and immunoblotted using the following antibodies: rabbit HA (Biolegend #902302) and rabbit myc (Proteintech #16286-1-AP). Bound primary antibodies were visualized using horseradish peroxidase-linked secondary antibodies (GE Healthcare), and immunoblot images were obtained using an ImageQuant LAS4000 Biomolecular Imager and analyzed using ImageQuant LAS4000 software (GE Life Sciences).

### Metabolic labeling with C15AlkOPP isoprenoid probe and click chemistry for fluorescence analysis

HEK293T cells were transfected with myc-tagged WT or SAAX mutant small GTPases. Ninety minutes following transfection, cells were metabolically labeled with 10 μM C15AlkOPP isoprenoid probe for 18 h. Cells were then harvested and lysed as described (58). Briefly, cell pellets were suspended and lysed by sonication in PBS + 1% SDS. Protein concentrations were determined using bicinchoninic acid Assay (Thermo Fisher Scientific, 23225), following the manufacturer’s protocol. Proteins (100 μg/100 μl) were subjected to click reaction with 25 μM TAMRA PEG_3_-N_3_ (BroadPharm), 1 mM Tris(2-carboxyethyl)phosphine hydrochloride (Sigma-Aldrich), 0.1 mM Tris[(1-benzyl-1H-1,2,3-triazol-4-yl)methyl]amine (Sigma-Aldrich), and 1 mM CuSO_4_ in the dark with gentle shaking for 1 h at room temperature. Proteins were precipitated using ProteoExtract Protein Precipitation Kit (Calbiochem, cat. no. 539180), following the manufacturer’s protocol. Resulting protein pellets were suspended in Laemmli sample buffer, boiled for 5 min, run on 12% SDS-PAGE gels, and transferred to PVDF. Fluorescence and immunoblotting images were obtained using Azure c600 and analyzed using AzureSpot software (Azure Biosystems). Fluorescence optical densities of overexpressed proteins were normalized to the optical density of myc-tagged protein.

### Immunofluorescence

HEK293T cells were plated on glass chamber slides and transfected with the indicated cDNAs encoding myc-tagged small GTPases. All cDNAs were transfected using Lipofectamine LTX with Plus Reagent (Life Technologies), according to the manufacturer’s protocol. Cells were fixed with 4% paraformaldehyde in PBS (15 min, on ice). After fixation, cells were incubated in a quench solution of 50 mM ammonium chloride (10 min, room temperature). Cells were then permeabilized with 0.2% Triton-X-100 in PBS (10 min, room temperature), blocked in 1% bovine serum albumin (BSA)/PBS (60 min, room temperature), and incubated with rabbit anti-myc antibody (Proteintech #16286-1-AP, 1:300) diluted in 1% BSA/PBS (60 min, room temperature). Following incubation with primary antibody, the cells were incubated with Alexa Fluor Plus 488 conjugated anti-rabbit secondary antibody (60 min, room temperature), incubated with 4′,6-diamidino-2-phenylindole solution (10 min, room temperature), mounted in mounting media (Invitrogen, #P36961), and imaged using a Nikon Eclipse Ni fluorescent microscope.

### Protein modeling and docking

Models for RAC1 (UniProt P63000-1) and RAC1B (P63000-2) were created using YASARA homology modeling, allowing the many solved structures to be merged in an unbiased approach. Docking of RAC1 or RAC1B to SmgGDS-558 (P52306-2) was performed using AutoDock (RAC1 docked to 5 ensembles of SmgGDS-558) or with RAC1 molecular replacement of solved structure 5zhx (RHOA-SmgGDS). Cluster analysis of both docking results in YASARA was done before energy minimization with the AMBER03 force field. The RAC1-SmgGDS was then used to model the RAC1B loop insertion with multiple starting conformations. MD simulations were performed on the models of RAC1 and the several different RAC1B conformations. Proteins were placed in a simulation box extended 5 Å from all atoms, pKa predictions for side chains set at pH 7.4, water at 0.997 g/ml added, and NaCl at 0.9% mass fraction added. The AMBER03 force field calculations were used for 20 ns of simulation, capturing atom positions every 25 picoseconds. Analysis of this simulation was performed, calculating global movement of carbon alphas at each time point and the average movement of each carbon alpha over the entire simulation. Dynamics cross correlation matrix analysis was done to correlate each amino acid with every other amino acid in the simulation. An extended simulation of the top RAC1B conformation was performed for 650 ns to confirm stable amino acid interactions.

### RAC activation assay

HEK293T cells were transfected with myc-tagged WT or SAAX mutant RAC1 or RAC1B, and 24 h later the activity of the expressed RAC1 or RAC1B was assessed by pulldown of the GTP-bound GTPase using a commercial assay according to the manufacturer’s protocol (Catalog #BK035, Cytoskeleton, Inc). Briefly, this assay utilizes the Rac-binding domain of activated kinase 1 as a GST fusion protein. This domain has been shown to bind specifically to the GTP-bound form of Rac proteins, which allows for pull down of GTP-bound Rac with glutathione affinity beads. Lysates and pull-down products were subjected to SDS-PAGE, transferred to PVDF membrane, and immunoblotted using mouse myc antibody (Catalog #60003-2-Ig, Proteintech). Optical densities of the pull-down products were normalized to the optical density of total myc-tagged protein in each lane.

### Statistical analysis

Statistical analysis was performed using GraphPad Prism 9. Data are presented as mean ± SD from three or more biological replicates. Statistical significance was determined using one-way ANOVA followed by Tukey’s or Dunnett’s multiple comparisons posthoc tests, or by paired, two-tailed Student *t* test, as indicated in the figure legend. Statistical significance was determined at *p* < 0.05.

## Data availability

Most of the data described are contained within the article. Any additional data or further description will be shared upon request to the corresponding author.

## Supporting information

This article contains supporting information.

## Conflict of interest

The authors declare that they have no conflicts of interest with the contents of this article.
