# Supplementary material for: GTPase splice variants RAC1 and RAC1B display isoform-specific differences in localization, prenylation, and interaction with the chaperone protein SmgGDS
Source: J Biol Chem. 2023 Apr 12;299(6):104698. doi: 10.1016/j.jbc.2023.104698 (PMC10206184; doi:10.1016/j.jbc.2023.104698)
Supplement: Supplemental Figures S1–S4 [file mmc1.docx]

Title: GTPase splice variants RAC1 and RAC1B display isoform-specific differences in localization, prenylation, and interaction with the chaperone protein SmgGDS

Authors: Olivia J. Koehn, Ellen Lorimer, Bethany Unger, Ra’Mal Harris, Akansha S. Das, Kiall F. Suazo, Shelby A. Auger, Mark D. Distefano, Jeremy W. Prokop, Carol L. Williams

Material Included:

Figure S1. Densitometry values of immunoblots shown in Fig. 2.

Figure S2. Densitometry values of immunoblot shown in Fig. 3.

Figure S3. Densitometry values of immunoblots shown in Fig. 9.


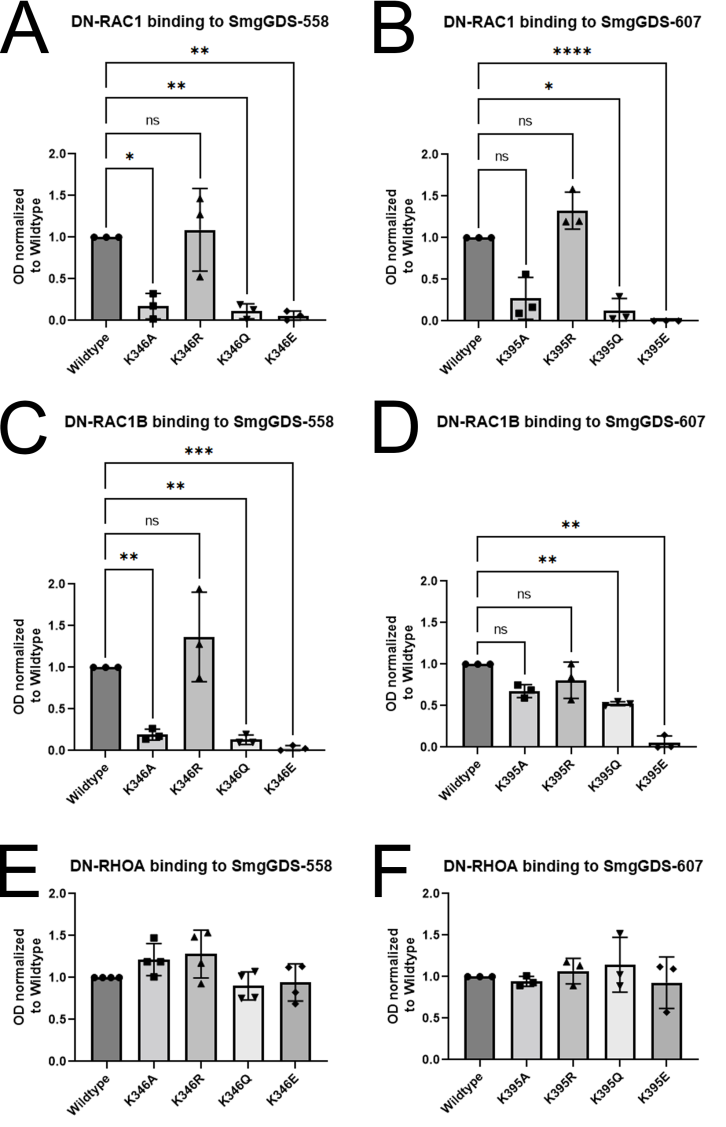


Fig. S1. Densitometry values of immunoblots shown in Fig. 2. The Optical Density (OD) of the small GTPase in the immunoprecipitate was normalized to the OD of SmgGDS in the immunoprecipitate. Graphed values depict the relative amount of (A and B) myc-DN-RAC1, (C and D) myc-DN-RAC1B, or (E and F) myc-DN-RHOA that co-precipitated with SmgGDS, normalized to the amount of small GTPase that co-precipitated with wildtype SmgGDS. Statistical significance was determined using one-way ANOVA followed by Dunnett’s multiple comparisons post-hoc test (*P<0.05, **P<0.01, ***P<0.001, ****P<0.0001, ns- not significant).


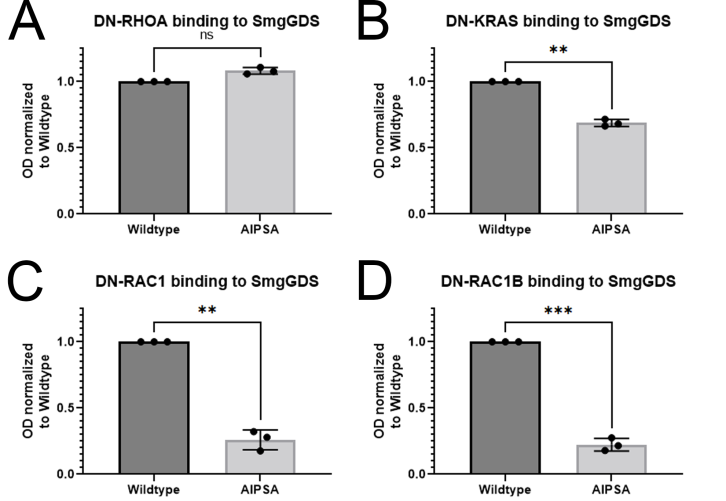


Fig. S2. Densitometry values of immunoblot shown in Fig. 3. The Optical Density (OD) of the small GTPase in the immunoprecipitate was normalized to the OD of SmgGDS in the immunoprecipitate. Graphed values depict the relative amount of (A) myc-DN-RHOA, (B) myc-DN-KRAS, (C) myc-DN-RAC1, or (D) myc-DN-RAC1B that co-precipitated with SmgGDS, normalized to the amount of small GTPase that co-precipitated with wildtype SmgGDS. Statistical significance was determined using paired, 2-tailed Student t test (**P<0.01, ***P<0.001, ns- not significant).


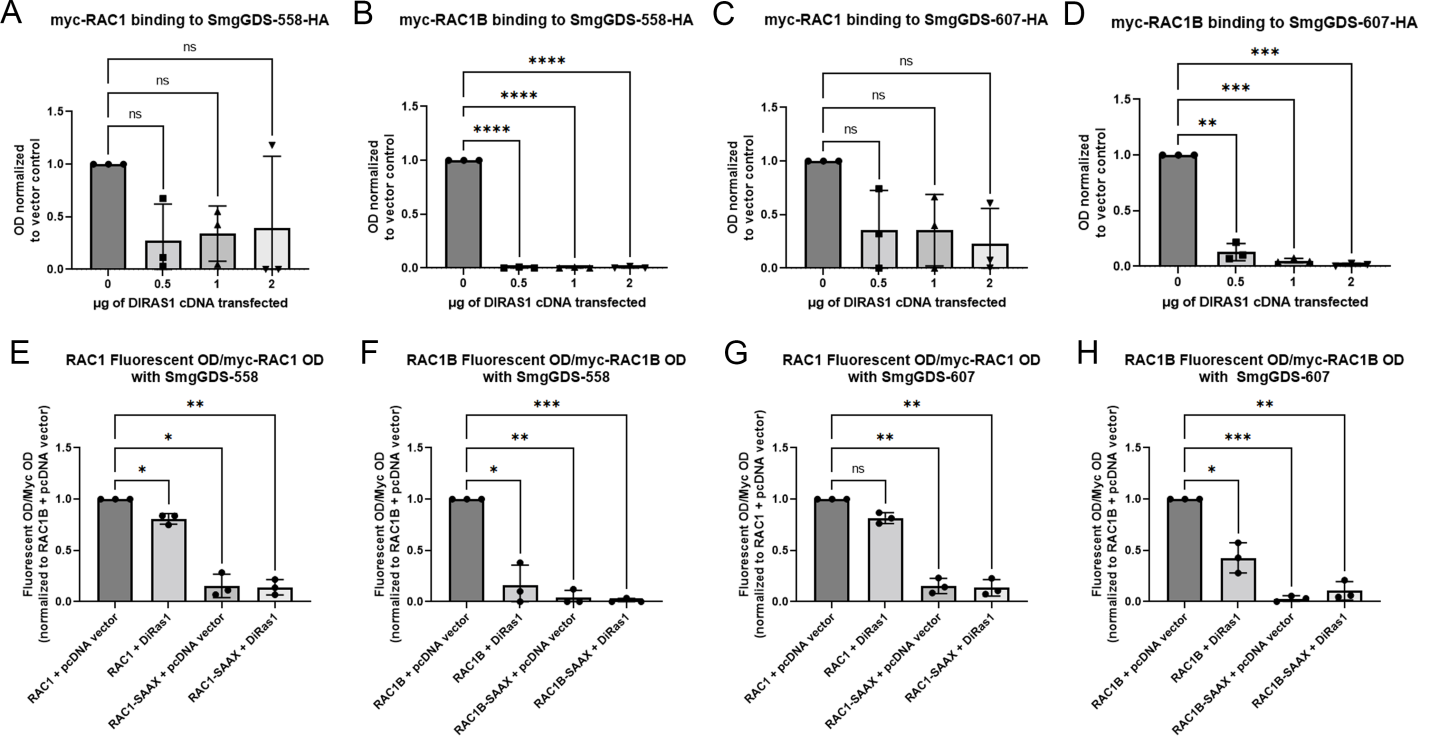


Fig. S3. Densitometry values of immunoblots shown in Fig. 9. (A-D) The Optical Density (OD) of the small GTPase in the immunoprecipitate was normalized to the OD of SmgGDS in the immunoprecipitate. Graphed values depict the relative amount of (A and C) myc-RAC1, (B and D) myc-RAC1B co-precipitated with SmgGDS, normalized to the amount of small GTPase that co-precipitated with SmgGDS in the absence of DIRAS1 expression. Statistical significance was determined using one-way ANOVA followed by Dunnett’s multiple comparisons post-hoc test (*P<0.05, **P<0.01, ***P<0.001, ****P<0.0001, ns- not significant). (E-F) Optical densities of fluorescent protein were normalized to optical densities of myc-tagged protein. Graphed values depict the relative amount of prenylated (E and G) myc-RAC1 or (F and H) myc-RAC1B, normalized to the amount of prenylated small GTPase in the absence of DIRAS1 expression. Statistical significance was determined using one-way ANOVA followed by Dunnett’s multiple comparisons post-hoc test (*P<0.05, **P<0.01, ***P<0.001, ns- not significant)
